# Alcohol use disorder and its association with quality of life and mortality in Chinese male adults: a population-based cohort study

**DOI:** 10.1186/s12889-022-13146-4

**Published:** 2022-04-19

**Authors:** Jiapeng Lu, Yang Yang, Jianlan Cui, Wei Xu, Chaoqun Wu, Jing Li, Xi Li

**Affiliations:** National Clinical Research Center for Cardiovascular Diseases, State Key Laboratory of Cardiovascular Disease, National Center for Cardiovascular Diseases, Fuwai Hospital, Chinese Academy of Medical Sciences and Peking Union Medical College, Beijing, People’s Republic of China

**Keywords:** Alcohol use disorder, Quality of life, Mortality

## Abstract

**Aims:**

We aimed to demonstrate the distribution of alcohol use disorder (AUD) in China and assess its association with quality of life and mortality.

**Methods:**

We studied 367 120 men aged 35–75 years from 31 provinces in the China Patient-Centered Evaluative Assessment of Cardiac Events (PEACE) Million Persons Project. At baseline, AUD was assessed by alcohol use disorders identification test score, and EQ-5D-3L questionnaire was used to measure the quality of life. Mortality data was collected via National Mortality Surveillance System and Vital Registration. Mixed models were fitted to assess the associations of AUD with quality of life, and Cox proportional hazard models were fitted for the associations with all-cause and cause-specific mortality.

**Results:**

We identified 39 163 men with AUD, which accounted for 10.7% of male participants and 25.8% of male drinkers. In the multivariable analysis, male drinkers who were aged 45–54 years, with higher education level, currently smoking, obese, with diagnosed hypertension, and without diagnosed cardiovascular diseases had higher rates of AUD. Male drinkers with AUD were less likely to have optimal QOL compared with those without AUD (OR: 0.63, 95% CI: 0.61–0.65, *P* < 0.001). Moreover, among male drinkers, AUD was prospectively associated with a 20% higher risk for all-cause mortality and a 30% higher risk for mortality from cancer.

**Conclusions:**

In China one fourth of men who drank alcohol had AUD, which was associated with lower QOL and higher risk of all-cause mortality. National policies or strategies are urgently needed to control alcohol-related harms.

**Supplementary Information:**

The online version contains supplementary material available at 10.1186/s12889-022-13146-4.

## Key Points

- Question: Is alcohol use disorder associated with quality of life and mortality in male drinkers?
- Findings: Among 152,514 male drinkers, alcohol use disorder was associated with a 40% lower probability of having optimal quality of life and prospectively associated with a 20% higher risk for all-cause mortality and a 30% higher risk for mortality from cancer.
- Meaning: Policies or strategies for reducing the affordability and accessibility of alcohol, encouraging behavioral intervention for alcohol drinkers and improving the accessibility to treatment for patients are urgently needed to prevent and control the alcohol-related harms.

## Introduction

Alcohol use caused 108 million (9%) disability adjusted life years (DALY) globally, of which one sixth were directly related to alcohol use disorder (AUD) [1]. AUD is one of the most prevalent mental disorders affecting nearly 10% of the general population worldwide [2, 3]. Understanding the population distribution and health effects of AUD is necessary for developing intervention strategies for reducing the related disease burden.

A systematic review including 38 studies published before 2010 has reported that the prevalence of AUD in China was 10.1% in men and 0.1% in women, but the quality of included studies was generally low and the between-study heterogeneity was very large (I^2^ > 99%) [4]. Other prior studies also evaluated the burden of alcohol abuse or problem drinking in some regions of China, and identified the potential risk factors of alcohol abuse, such as middle age, male gender and low education level; however, these findings were constrained by small sample sizes and incomplete regional coverage [5–7]. Despite the increasing alcohol consumption and growing proportion of heavy drinking in China [8, 9], national epidemiological evidence on AUD is still limited. Previous studies have revealed that male drinkers with AUD or problem drinking had about a two-fold higher risk of all-cause mortality, but the specific associations of AUD with cause-specific mortality, such as mortality from cardiovascular diseases, cancer and injuries, were not reported [6, 10, 11]. Another study found that males with problem drinking had poorer self-reported health and were more likely to have symptoms of depression and anxiety, while little is known about how quality of life was compromised in male with AUD [6].

Accordingly, we aimed to demonstrate the distribution of AUD in men and its variations across population subgroups in China, identify potential sociodemographic and health-related characteristics associated with AUD, and assess the associations of AUD with quality of life and all-cause and cause-specific mortality.

## Methods

### Study design and population

The China PEACE Million Persons Project is a government-funded public health project across China. The details of the project design have been described previously [12]. Briefly, from September 2014 through November 2019, 254 county-level regions in all 31 provinces in mainland China were selected as study sites to provide diversity in geographic distribution, population structure (ethnicity distribution), and exposure to risk factors and disease patterns. Although the study sites were not randomly selected, the selection had considered population size, population stability, and local capacity to support the project. Local residents aged 35 to 75 years, who were currently registered in the community’s Hukou (a record officially identifying area residents), or had lived in the community for at least 6 of the prior 12 months, were invited and recruited in this project. The overall response rate was about 30%. Enrolled participants with serial project ID number ended with 1, 3, 5, or 7, who were chosen for detailed lifestyle survey as a representative sample of the entire project cohort, were included in the study analyses. We excluded female participants as the rate of alcohol drinking among them was very low (approximately 5.9%). The central ethics committee at the China National Center for Cardiovascular Diseases approved this project. All enrolled participants provided written informed consent.

### Data collection and variables

For each participant, standardized in-person interviews were conducted by trained personnel to collect information on socio-demographic status (education, annual household income, health insurance and marital status), lifestyle (tobacco smoking, alcohol drinking, diet and physical activity), medical history and quality of life. Blood pressure, height, and weight were measured using the unified protocols and devices.

The presence of AUD was evaluated by the alcohol use disorders identification test (AUDIT) score [13]. Each of the 10 questions in the AUDIT has a set of responses, which has a score ranging from 0 to 4. The response scores of all 10 questions were added up to get the total score for each participant. The presence of AUD was defined as the total AUDIT score equal to or greater than 8 according to the recommendation by the World Health Organization (WHO) [13]. Current drinker was defined based on Question 1, as drinking at least once every month during the past 12 months [14]. The AUDIT has three conceptual domains including hazardous alcohol use, presence of dependence symptoms and harmful alcohol use. A score of 1 or more on Question 2 or Question 3 indicates consumption at a hazardous level. Points scored above 0 on questions 4–6 (especially weekly or daily symptoms) imply the presence or incipience of alcohol dependence symptoms. Points scored on questions 7–10 indicate that harmful alcohol use has already occurred. The AUDIT has been validated in Chinese population, which has high validity (0.93–0.95) and high reliability (0.95–0.99) [15]. And it has high sensitivity (0.877–1.000) and specificity (0.881–0.900) for identifying participants with AUD.

The three-level EuroQol five-dimension (EQ-5D-3L) instrument was used to assess the quality of life (QOL) [16]. For each participant, an index score from the EQ-5D-3L instrument was calculated based on the time trade-off method for Chinese population [17]. The highest index score was 0.961 as the optimal QOL, indicating no health-related reduction of QOL. A lower index score indicated a lower QOL.

Further, we ascertained the vital status and causes of death of each enrolled participant through China’s Centre for Disease Prevention and Control’s National Mortality Surveillance System and Vital Registration, with annual active confirmation from local residential, medical, health insurance and administrative records. We used the International Classification of Diseases (ICD)-10 to code the mortality records. All-cause mortality and mortality from CVD (ICD-10: I00-I99), cancer (ICD10: C00-C97) and injuries (ICD10: L55-L55.9, L56.3, L56.8-L56.9, L58-L58.9, U00-U03, V00-V86.9, V87.2-V87.3, V88.2-V88.3, V90-V98.8, W00-W46.2, W49-W62.9, W64-W70.9, W73-W75.9, W77-W81.9, W83-W94.9, W97.9, W99-X06.9, X08-X39.9, X46-X48.9, X50-X54.9, X57-X58.9, X60-X64.9, X66-Y08.9, Y35-Y84.9, Y87.0-Y87.1, Y88-Y88.3, Y89.0-Y89.1) were analyzed.

### Statistical analyses

Patient characteristics were summarized using proportion for categorical variables and mean ± standard deviation or median (interquartile range) as appropriate for continuous variables. Continuous variables were compared using the Student’s t test or Mann–Whitney U test according to data distribution, and categorical variables using the chi-square test. Standardized mean differences (SMD) were calculated to compare patient characteristics between current drinkers and non-drinkers, and between drinkers with and without AUD. When the absolute value of the SMD was < 0.2, the between-group difference was thought to be small [18]. The 95% confidence intervals (CI) for prevalence rates were calculated using the Clopper-Pearson method [19]. To quantify the variability in rates of AUD among the study sites, we calculated the median odds ratio (MOR) by fitting a multivariable mixed model with random effects at the study site level.

We fitted multivariable mixed models with study site as random effects and a logit link function to assess the associations of demographic, socioeconomic and health-related factors with AUD in the study population. The four dependent variables were whether the male drinkers had AUD, hazardous alcohol use, dependence symptoms, and harmful alcohol use. The individual characteristics included in the model were age, urbanity, education level, annual household income, marital status, smoking, obesity, and diagnosed CVD, hypertension, and diabetes.

We assessed the association of AUD with quality of life and mortality in all male drinkers and in subgroups stratified by age group and geographical region. A multivariable mixed models with study sites as random effects and a logit link function were fitted to assess the associations of AUD with quality of life (optimal QOL vs. non-optimal QOL) adjusted for age, geographic regions, education level, annual household income, marital status, medical insurance, smoking, history of hypertension, history of diabetes and BMI groups. Cox proportional hazard models were used to calculate hazard ratios (HR) and 95% CIs for the AUD with all-cause mortality. Competing risk models were used to calculate HRs and 95% CIs for the AUD with cause-specific mortality. All models were adjusted by age, education, annual household income, current smoking and BMI. As the mortality data were available up to 31 December 2019, we censored the follow-up at this date or the date of death, whichever occurred first.

*P* < 0.05 was considered statistically significant. All analyses were conducted with SAS 9.4 (SAS Institute Inc., Cary, North Carolina).

## Results

### Participant characteristics

Among the 367 120 men included, the average age was 56.6 ± 10.1 years. Overall, about three fifths of them were living in rural areas, 19% had an annual household income over 50 000 Yuan RMB, 26% had a high school education or above, 98% had social health insurance, and 95% were currently in marriage (Table 1). Meanwhile, nearly half of these men were current smokers, one quarter with low physical activity, and one sixth obese. Regarding the medical comorbidities, 25% of them had diagnosed hypertension, 7.6% diagnosed diabetes, 4.0% prior coronary health disease or stroke, 0.2% chronic kidney disease, and 0.1% cancers (Table 1).

**Table 1:** Basic characteristics of male participants by drinking status

	**All male**	**Male drinkers**	**Male with AUD**
*N*	367,120	152,514	39,163
Mean of age, mean (SD)	56.64 (10.05)	55.65 (9.76)	55.1 (9.42)
Age (years)			
35–44	51,170 (13.9)	22,709 (14.9)	5818 (14.9)
45–54	104,494 (28.5)	48,133 (31.6)	13,357 (34.1)
55–64	116,643 (31.8)	49,161 (32.2)	12,798 (32.7)
65–75	94,813 (25.8)	32,511 (21.3)	7190 (18.4)
Urbanity			
Urban	140,965 (38.4)	57,503 (37.7)	14,281 (36.5)
Rural	226,155 (61.6)	95,011 (62.3)	24,882 (63.5)
Geographical regions			
Eastern	133,796 (36.4)	58,658 (38.5)	15,321 (39.1)
Central	109,069 (29.7)	46,370 (30.4)	12,120 (30.9)
Western	124,255 (33.8)	47,486 (31.1)	11,722 (29.9)
Education			
Primary school or lower	132,932 (36.2)	50,709 (33.2)	12,999 (33.2)
Middle school	133,343 (36.3)	56,994 (37.4)	14,825 (37.9)
High school	62,547 (17)	26,786 (17.6)	6690 (17.1)
College or above	34,421 (9.4)	16,682 (10.9)	4374 (11.2)
Unknown	3877 (1.1)	1343 (0.9)	275 (0.7)
Household Income (Yuan RMB/year)			
< 10 000	63,156 (17.2)	23,968 (15.7)	6153 (15.7)
10 000–50 000	202,665 (55.2)	85,491 (56.1)	21,915 (56)
> 50 000	70,469 (19.2)	32,223 (21.1)	8479 (21.7)
Unknown	30,830 (8.4)	10,832 (7.1)	2616 (6.7)
Marital Status			
Married	348,525 (94.9)	145,360 (95.3)	37,380 (95.4)
Widowed, separated, divorced, single	14,987 (4.1)	5880 (3.9)	1506 (3.8)
Unknown	3608 (1.0)	1274 (0.8)	277 (0.7)
Health Insurance Status			
Insured	360,660 (98.2)	150,203 (98.5)	38,658 (98.7)
Uninsured	2163 (0.6)	885 (0.6)	214 (0.5)
Unknown	4297 (1.2)	1426 (0.9)	291 (0.7)
Risk factors			
Current smoker	170,245 (46.4)	89,745 (58.8)*	25,690 (65.6)
Low physical activity	91,262 (24.9)	33,989 (22.3)	7902 (20.2)
Obesity	60,862 (16.6)	27,765 (18.2)	8048 (20.6)
Medical history			
Hypertension	90,775 (24.7)	39,910 (26.2)	11,541 (29.5)
Diabetes	28,053 (7.6)	11,285 (7.4)	2975 (7.6)
Cardiovascular diseases	14,829 (4)	5090 (3.3)	1200 (3.1)
Chronic kidney diseases	881 (0.2)	329 (0.2)	114 (0.3)
Cancers	394 (0.1)	99 (0.1)	34(0.1)

^*^ The absolute value of standardized mean difference is larger than 0.2, indicating the difference between current drinkers and non-drinkers is significant. *AUD* Alcohol use disorder, *SD* Standard deviation

A total of 152 514 (41.5%) men in the study cohort were current drinkers, and their average age was 55.7 ± 9.8 years. The socioeconomic profiles, risk factors and medical histories of current drinkers were similar to those of non-drinkers (Table 1).

### Prevalence of alcohol use disorder

The prevalence of AUD was showed in Table 2. The crude rate of AUD in the study population was 10.7% (39 163, 95%CI: 10.6%-10.8%). After age-standardization according to the 2010 population census of China, the rate of AUD was 11.4% (95%CI: 11.3%-11.5%). Across the 247 study sites with at least 200 men per site, the age-standardized AUD rates ranged from 0.2% to 35.1%, with a median of 9.8% and an MOR of 2.2 (95%CI: 2.0–2.3). Among the male drinkers, the crude rate of AUD was 25.6% (95%CI: 25.4%-25.8%). After age-standardization according to the 2010 population census of China, the rate of AUD was 26.1% (95%CI: 25.9%-26.4%). Across the 220 study sites with at least 200 male drinkers per site, the proportion of male drinkers with AUD ranged from 3.3% to 57.7% across study sites, with a median of 23.1% and an MOR of 1.8 (95%CI: 1.7–1.9).

**Table 2:** Prevalence of AUD among all male participants and male drinkers

	***N***	**Crude rates** %, 95% CI	**Age-standardized rates** %, 95% CI
**All male participants**			
AUD	39,163	10.7, 10.6–10.8	11.4, 11.3–11.5
hazardous alcohol use	65,259	17.8, 17.7–17.9	20.0, 19.9–20.1
harmful alcohol use	68,639	18.7, 18.6–18.8	19.7, 19.6–19.8
dependence symptoms	12,318	3.4, 3.3–3.4	3.9, 3.8–3.9
**Male drinkers**			
AUD	39,068	25.6, 25.4–25.8	26.1, 25.9–26.4
hazardous alcohol use	65,259	42.8, 42.5–43.0	45.9, 45.7–46.2
harmful alcohol use	61,410	40.3, 40.0–40.5	41.2, 41.0–41.5
dependence symptoms	12,315	8.1, 7.9–8.2	8.9, 8.8–9.1

*AUD* Alcohol use disorder, *CI* Confidence interval

Among male drinkers, 42.8% of them had hazardous alcohol use, followed by 40.3% having harmful alcohol use and 8.1% with dependence symptoms. After age-standardization, the rate was 45.9%, 41.2%, and 8.9%, respectively. The proportion of male drinkers experiencing hazardous alcohol use ranged from 10.9% to 88.4% across study sites, with an MOR of 1.8 (95%CI: 1.7–1.9). And the proportion of male drinkers experiencing harmful alcohol use ranged from 5.7% to 78.6% across study sites, with an MOR of 1.8 (95%CI: 1.7–1.9). The proportion of male drinkers having dependence symptoms ranged from 0.8% to 36.8% across study sites, with an MOR of 2.0 (95%CI: 1.8–2.1).

### Factors associated with alcohol use disorders

It was found that male drinkers who were aged 45–54 years, currently smoking, obese, with a higher education level, with diagnosed hypertension, and without diagnosed CVD had a higher rate of AUD (all *P* < 0.05) (Table 3). In the multivariable models, these correlations remained statistically significant.

**Table 3:** Association of individual characteristics with AUD among male drinkers

	**Prevalence of AUD** *N (%)*	**Adjusted OR (95% CI)**	***P* value**
Age (years)			< 0.001
35–44	5805 (25.6)	1.00	
45–54	13,334 (27.8)	1.07 (1.03–1.11)	
55–64	12,766 (26.0)	0.97 (0.93–1.01)	
65–75	7163 (22.0)	0.78 (0.74–0.82)	
Urbanity			0.35
Urban	14,247 (24.8)	1.00	
Rural	24,821 (26.2)	1.07 (0.92–1.23)	
Geographical regions			0.84
Eastern	15,321 (26.1)	1.00	
Central	12,120 (26.1)	1.01 (0.85–1.21)	
Western	11,722 (24.7)	1.01 (0.85–1.19)	
Education Level			0.004
Primary school or lower	12,959 (25.6)	1.00	
Middle school	14,796 (26.0)	1.00 (0.97–1.04)	
High school	6672 (25.0)	1.01 (0.97–1.05)	
College or above	4367 (26.2)	1.10 (1.05–1.16)	
Household Income (Yuan RMB/year)			0.06
< 10 000	6134 (25.6)	1.00	
10 000–50 000	21,861 (25.6)	1.00 (0.97–1.04)	
> 50 000	8463 (26.2)	1.03 (0.99–1.09)	
Marital Status			0.15
Widowed, separated, divorced, single	1503 (25.6)	1.00	
Married	37,289 (25.6)	1.04 (0.97–1.11)	
Current smoker			< 0.001
No	13,422 (21.4)	1.00	
Yes	25,646 (28.6)	1.53 (1.49–1.57)	
Obesity			< 0.001
No	31,035 (24.8)	1.00	
Yes	8033 (29.0)	1.17 (1.14–1.21)	
Diagnosed CVD			0.01
No	37,869 (25.6)	1.00	
Yes	1199 (23.6)	0.91 (0.84–0.97)	
Diagnosed hypertension			< 0.001
No	27,554 (24.4)	1.00	
Yes	11,514 (28.8)	1.26 (1.22–1.29)	
Diagnosed diabetes			0.36
No	36,103 (25.6)	1.00	
Yes	2965 (26.2)	1.02 (0.97–1.07)	

*AUD* Alcohol use disorder, *CVD* Cardiovascular diseases, *OR* Odds ratio, *CI* Confidence interval

Regarding different conceptual domains of AUD, for hazardous alcohol use, dependence symptoms and harmful alcohol use, the associated factors including age, education, smoking, obesity and diagnosed hypertension were generally similar with the overall AUD (Table 4). For dependence symptoms, the males living in rural areas had a higher rate of dependence symptoms than those in urban areas (OR: 1.27, 95%CI: 1.06–1.52).

**Table 4:** OR and 95% CI for the association of individual characteristics with the three domains of AUD among male drinkers

	**Hazardous alcohol use**	**Dependence symptoms**	**Harmful alcohol use**
Age (years)			
35–44	1.00	1.00	1.00
45–54	0.97 (0.93–1.00)	0.96 (0.91–1.02)	1.06 (1.02–1.09)
55–64	0.74 (0.71–0.77)	0.78 (0.73–0.83)	0.97 (0.94–1.01)
65–75	0.52 (0.50–0.55)	0.55 (0.51–0.59)	0.86 (0.83–0.90)
Urbanity			
Urban	1.00	1.00	1.00
Rural	1.10 (0.95–1.29)	1.27 (1.06–1.52)	1.03 (0.89–1.20)
Education			
Primary school or lower	1.00	1.00	1.00
Middle school	1.04 (1.01–1.07)	0.98 (0.93–1.03)	1.06 (1.03–1.09)
High school	1.11 (1.07–1.15)	1.01 (0.94–1.07)	1.11 (1.07–1.16)
College or above	1.25 (1.19–1.31)	1.10 (1.02–1.19)	1.20 (1.15–1.26)
Household Income (RMB Yuan/year)			
< 10 000	1.00	1.00	1.00
10 000–50 000	1.07 (1.03–1.10)	0.98 (0.93–1.04)	0.99 (0.96–1.02)
> 50 000	1.12 (1.07–1.17)	1.04 (0.97–1.13)	1.04 (1.00–1.09)
Marital Status			
Widowed, separated, divorced, single	1.00	1.00	1.00
Married	1.03 (0.97–1.09)	0.97 (0.87–1.07)	1.06 (1.00–1.12)
Current smoker			
No	1.00	1.00	1.00
Yes	1.48 (1.44–1.51)	1.41 (1.35–1.47)	1.36 (1.32–1.39)
Obesity			
No	1.00	1.00	1.00
Yes	1.21 (1.18–1.25)	1.14 (1.09–1.20)	1.14 (1.11–1.17)
Diagnosed CVD			
No	1.00	1.00	1.00
Yes	0.78 (0.74–0.84)	0.95 (0.85–1.07)	1.09 (1.02–1.16)
Diagnosed hypertension			
No	1.00	1.00	1.00
Yes	1.15 (1.12–1.18)	1.22 (1.16–1.28)	1.24 (1.21–1.28)
Diagnosed diabetes			
No	1.00	1.00	1.00
Yes	0.96 (0.92–1.01)	1.03 (0.95–1.11)	1.12 (1.08–1.17)

*AUD* alcohol use disorder, *CVD* cardiovascular diseases, *OR* odds ratio, *CI* confidence interval

### Association of AUD with mortality and quality of life

**Fig. 1:**
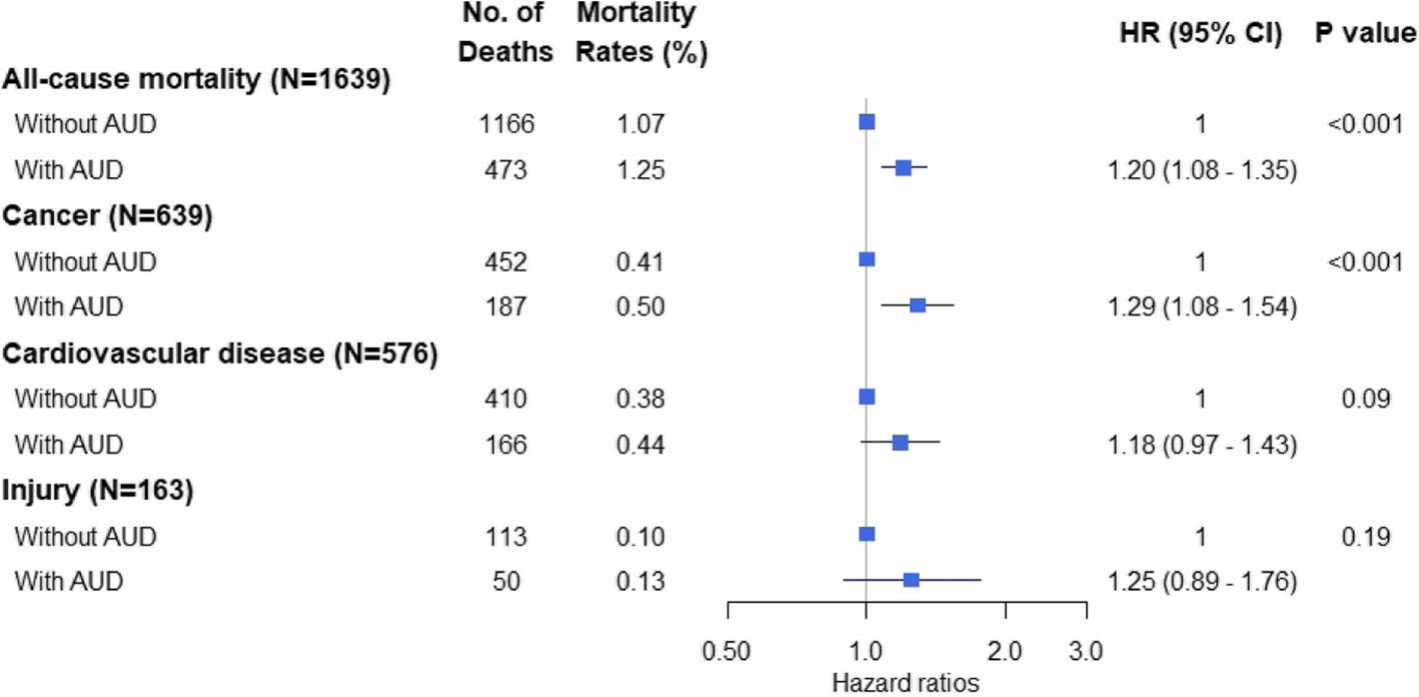
Association of AUD with all-cause and cause-specific mortality among male drinkers. *AUD* Alcohol use disorder, *HR* Hazard ratio, *CI* Confidence interval

During the follow-up with a median duration of 2.4 years (IQR: 1.2–3.6 years), there were 1639 (1.12%) deaths occurring among male drinkers, including 575 (0.39%) deaths from CVD, 639 (0.43%) from cancer, and 163 (0.11%) from injuries. All-cause and cause-specific mortality rates were both higher in male drinkers with AUD compared with those without AUD. After adjusting covariates in the Cox regression model, male drinkers with AUD had a 20% higher risk for all-cause mortality compared with those without AUD (HR: 1.20, 95% CI: 1.08–1.35, *P* < 0.001). Regarding the specific causes of death, male drinkers with AUD had a 30% higher risk for mortality from cancer (HR: 1.29, 95% CI: 1.08–1.54, *P* = 0.004) compared with those without AUD; while no significant association was found for mortality from CVD (HR: 1.18, 95% CI: 0.97–1.43, *P* = 0.09) or injuries (HR: 1.25, 95%CI: 0.89–1.76, *P* = 0.19) (Fig. 1). Subgroup analyses showed that the AUD was significantly associated with higher risk for all-cause mortality in the central (HR: 1.55, 95% CI: 1.20–2.01) and western (HR: 1.37, 95% CI: 1.07–1.76) regions, but not in eastern region (Additional file 1). And AUD was significantly associated with higher risk for cancer mortality in the central region (HR: 1.85, 95% CI: 1.21–2.81), but not in other regions.

**Fig. 2:**
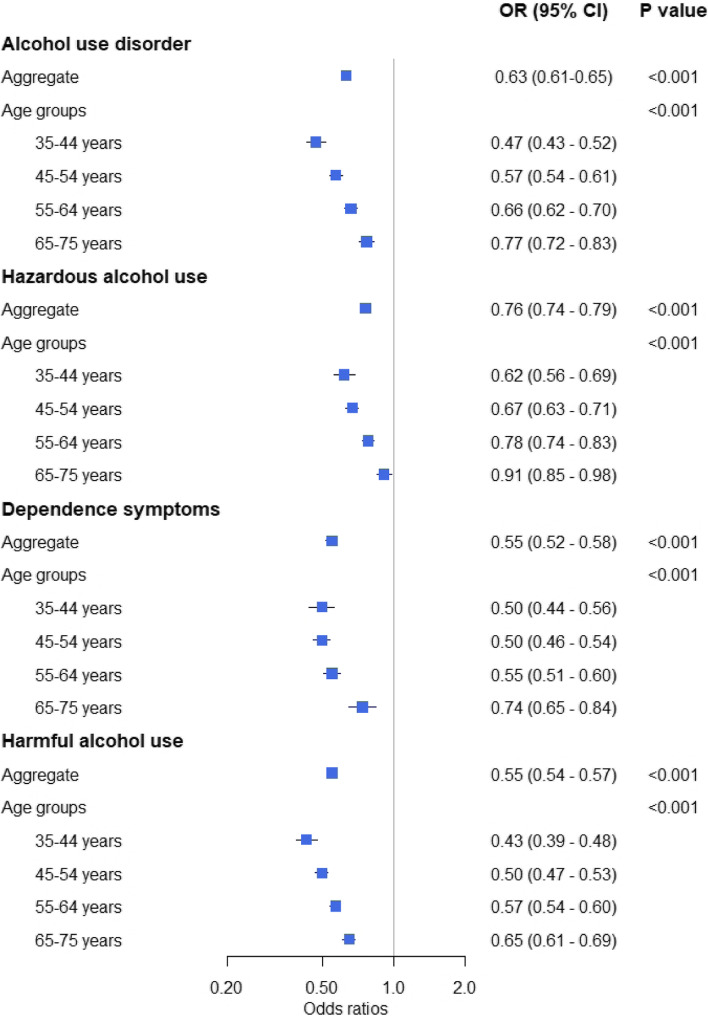
Association of AUD and its three domains with quality of life among male drinkers. *AUD* Alcohol use disorder, *OR* Odds ratio, *CI* Confidence interval

Based on the index score from EQ-5D-3L instrument, male drinkers with AUD had a slightly lower QOL compared with those without AUD (mean ± SD: 0.95 ± 0.04 for participants without AUD and 0.94 ± 0.05 for participants with AUD, P < 0.001). The crude rates of men with optimal QOL were 81.8% in those with AUD and 88.2% in those without AUD (OR: 0.65, 95% CI: 0.63–0.67, *P* < 0.001). After adjusting for individual characteristics, multivariable mixed models identified that male drinkers with AUD were less likely to have optimal QOL, compared with those without AUD (OR: 0.63, 95% CI: 0.61–0.65, *P* < 0.001). In addition, hazardous alcohol use (OR: 0.76, 95% CI: 0.74–0.79, *P* < 0.001), dependence symptoms (OR: 0.55, 95% CI: 0.52–0.58, *P* < 0.001), and harmful alcohol use (OR: 0.55, 95% CI: 0.54–0.57, *P* < 0.001) were significantly associated with optimal QOL. Subgroup analyses showed that the associations of AUD and its three domains with QOL were significant in all age groups, and the magnitude of the associations was larger in those who were younger (Fig. 2). Significant associations of AUD and its three domains with QOL were also identified in all geographical regions (Additional file 1).

## Discussion

In this national population-based study, we found that around one fourth of male drinkers (i.e., nearly one ninth of male adults) in China had AUD based on AUDIT score. Male drinkers who were aged 45–54 years, currently smoking, obese, with a higher education level, with diagnosed hypertension, or without diagnosed CVD had higher rates of AUD. The presence of AUD was associated with lower QOL as well as higher risk of all-cause mortality and cancer-specific mortality.

This study adds to the existing literature in several ways. First, to our knowledge, this is the first time that a validated screening tool has been used to estimate the prevalence of AUD in China at the national level. The rate of AUD was consistent with the findings from a systematic review in China (10.1% in male) using heterogeneous tools [4] and a population-based study on problem drinking (8% of male and 24% of male drinkers) [6]. The overall rate of AUD in China was lower than that in the United States. A national survey in the US reported that about 18% of male adults had AUD [20]. In our study, male drinkers aged 45-54 had the highest rate of AUD, which could be due to the highest prevalence of regular drinking and alcohol consumption in this age group [21]. Inconsistent with the previous study [6], we found male drinkers with a higher education level had a slightly higher rate of AUD. This could be explained by the more rapid increase in prevalence of alcohol drinking and higher percentage of heavier drinkers among Chinese men with a higher education level in the past decade [8]. Moreover, this study recruited participants with diverse sociodemographic characteristics, who came from 55% of the prefecture-level cities scattered over all 31 provinces of mainland China. It is the first time a 20-fold difference in the prevalence of AUD at county level was reported, which awaits further studies to explore the potential causes of alcohol problems in different regions of China.

Second, we described the prevalence of the three conceptual domains of AUD including hazardous alcohol use, presence of dependence symptoms and harmful alcohol use, and examined their associated individual characteristics. Compared with the presence of dependence symptoms, hazardous alcohol use and harmful alcohol use were more common in Chinese male drinkers, which was consistent with previous studies [22, 23]. Multivariable mixed models found that the individual characteristics associated with the three domains were largely similar with those of the overall AUD; meanwhile, living in rural areas was one of the most significant factors that were associated with a higher prevalence of dependence symptoms. A previous Chinese study with a regional representative sample also reported that rural residents were more likely to have alcohol dependence compared to urban residents [23]. The higher prevalence of dependence symptoms in rural areas was attributed to their higher level of alcohol consumption.

Thirdly, we explored the association of the presence of AUD with health outcomes including mortality and health-related QOL among male drinkers. Previous studies had reported an increasing risk of all-cause mortality with AUD or problem drinking [6, 10, 24]. Our study further revealed an even stronger association of AUD with cancer-specific mortality. The evidence of association of AUD with overall health status is limited, except for some mental disorders, such as anxiety and depression [6, 20, 25]. In our study, we investigated the relationship between AUD and health-related QOL, and observed that male drinkers with AUD were less likely to have optimal QOL compared with those without AUD. Further studies that could generate more detailed knowledge on the influence of AUD on different dimensions of QOL, such as functional and psychologic states, are needed. Moreover, we found that the impacts of AUD on QOL were stronger in younger population. Given the higher rate of AUD in younger group, more targeted and stronger strategies for middle age male drinkers should be considered to prevent AUD and the consequent health-related harms.

The findings based on our analyses have notable policy implications. Alcohol use was estimated to account for 2.84 million deaths annually, with an increase of 10% during the past decade [1]. In China, alcohol consumption per capita increased from 4.1 L in 2005 to 7.2 L in 2016, and the prevalence of heavy episodic drinking reached 53% among male drinkers [26]. Consequently, the prevalence of AUD and its health harm are likely to increase in the future. Health education and counselling for alcohol drinkers, which could effectively reduce alcohol consumption, should therefore be provided in primary care settings in order to prevent cardiovascular diseases and cancer [27]. Behavioral interventions for alcohol drinkers should also be included in the scopes of service in primary health care institutions. Secondly, although there are tertiary psychiatric hospitals with addiction units for the treatment of patients with AUD in several mega cities in China [28], the accessibility to the treatment by psychiatric specialists is yet to improve in most areas across the country.

The findings should be explained considering several limitations. First of all, the AUDIT is a screening tool, not a clinical diagnosis tool; thus, participants with AUD defined in this study were not clinically diagnosed. However, the AUDIT is an efficient tool for early detecting patients experiencing alcohol abuse and dependence in the primary care settings and understanding the burden of AUD in the general population [15]. Second, the QOL findings were derived from cross-sectional data. Third, the participants were followed up for a short period. The limited number of events may result in reduced statistical power to detect significant impact of AUD on mortality, especially on cause-specific mortality. Lastly, the study sample is not a nationally or regionally random sample, which would limit the representativeness of the rate of AUD observed in this study. Nevertheless, the association between AUD and health-related outcomes could still be generalizable to the general population.

In conclusion, our results showed that one in nine middle-aged men had AUD in China. Male drinkers with AUD were associated with lower QOL and higher risks of all-cause mortality and cancer-specific mortality. National strategies for reducing alcohol consumption and improving health education for alcohol drinkers in primary care settings are imperative to prevent and control alcohol-related harms.

### Summary box

#### What is already known on this topic

AUD or problem drinking was associated with some health-related measures, such as anxiety and depression.

Male drinkers with AUD or problem drinking had higher risk for all-cause mortality.

### What this study adds

Male drinkers with AUD were less likely to have optimal quality of life compared with those without AUD.

AUD was prospectively associated with a 20% higher risk for all-cause mortality and 30% higher risk for mortality from cancer.

## Supplementary Information

**Additional file 1.** Additional Table 1

## Data Availability

The datasets generated and/or analysed during the current study are not publicly available due to that the China PEACE Million Persons Project is a national program, and as the government policy stipulates, it is not permissible for the researchers to make the raw data publicly available at this time. And currently, it is not yet possible for other researchers to apply for the access.
