# Supplementary material for: Alcohol use disorder and its association with quality of life and mortality in Chinese male adults: a population-based cohort study
Source: BMC Public Health. 2022 Apr 19;22:789. doi: 10.1186/s12889-022-13146-4 (PMC9017962; doi:10.1186/s12889-022-13146-4)
Supplement: Supplementary file 1 — Additional file 1. Additional Table 1 [file 12889_2022_13146_MOESM1_ESM.pdf]

Additional Table 1. Association of AUD and all-cause and cause-specific mortality among male drinkers stratified by geographical regions

	All-cause mortality		Cancer		Cardiovascular disease		Injury	
	HR (95% CI)	P value	HR (95% CI)	P value	HR (95% CI)	P value	HR (95% CI)	P value
**Geographic regions**		0.005		0.31		0.19		0.23
Eastern	0.83 (0.63-1.10)		0.82 (0.54-1.24)		0.94 (0.59-1.50)		0.88 (0.34-2.28)	
Central	1.55 (1.20-2.01)		1.85 (1.21-2.81)		1.36 (0.87-2.12)		1.90 (0.94-3.84)	
Western	1.37 (1.07-1.76)		0.99 (0.61-1.59)		1.43 (0.95-2.16)		1.85 (0.95-3.61)	

AUD: alcohol use disorder, HR: hazard ratio; CI: confidence interval.

Additional Table 2. Association of AUD and its three domains with quality of life among male drinkers stratified by geographical regions

	AUD		Hazardous alcohol use		Dependence symptoms		Harmful alcohol use	
	OR (95% CI)	P value	OR (95% CI)	P value	OR (95% CI)	P value	OR (95% CI)	P value
**Geographic regions**		0.28		0.38		0.21		0.38
Eastern	0.64 (0.60-0.68)		0.76 (0.72-0.81)		0.54 (0.49-0.59)		0.55 (0.52-0.58)	
Central	0.64 (0.60-0.68)		0.74 (0.69-0.78)		0.54 (0.50-0.59)		0.58 (0.55-0.62)	
Western	0.61 (0.58-0.65)		0.80 (0.75-0.84)		0.57 (0.53-0.62)		0.53 (0.50-0.56)	

AUD: alcohol use disorder, OR: odds ratio; CI: confidence interval.
